# Exogenous IL-2 Controls the Balance in Th1, Th17, and Treg Cell Distribution in Patients with Progressive Rheumatoid Arthritis Treated with TNF-Alpha Inhibitors

**DOI:** 10.1007/s10753-014-9987-x

**Published:** 2014-08-22

**Authors:** Agata Kosmaczewska, Lidia Ciszak, Jerzy Swierkot, Aleksandra Szteblich, Katarzyna Kosciow, Irena Frydecka

**Affiliations:** 1 Department of Immunopathology, Institute of Immunology and Experimental Therapy, Polish Academy of Sciences, R. Weigla St. 12, 53-114 Wroclaw, Poland; 2 Department of Rheumatology and Internal Medicine, Wroclaw Medical University, Wroclaw, Poland; 3 Department of Hematology, Regional Hospital, Opole, Poland

**Keywords:** rheumatoid arthritis, MTX, iTNF, *in vitro* stimulation, rIL-2

## Abstract

Interleukin-2 (IL-2) has been suggested to control Treg/Th17 balance. Recently, we reported a relationship of rheumatoid arthritis (RA) activity/progression with irreversible systemic Treg and Th1 defects including serum IL-2 shortage. Herein, we explore the role of *in vitro* stimulation with rIL-2 in the observed immune alterations reversal. Patients with stable or progressive RA were assigned to methotrexate (MTX) group or to TNF-alpha inhibitors (iTNF) group, respectively. Flow cytometric analyses were performed before and after 6 months of treatment. Circulating Th1, Th17, and Treg cells were determined before and after 72-h culture with anti-CD3 + rIL-2. Before therapy, 72-h stimulation restored recently observed phenotypic Th cell alterations, except for the enriched Th17 subset normalized as late as after therapy in all patients. Under 6-month therapy, anti-CD3 stimulation changed the Th cell distribution only in progressive RA; despite Th1 enrichment, it revealed Treg population defects, which were completely reversed by exogenous IL-2 added to the stimulating culture. Our paper shows that in aggressive RA patients exhibiting serum IL-2 shortage despite iTNF therapy, exogenous rIL-2 is capable of promoting Treg differentiation affected by chronic activation, thus supporting its use in the combined strategy of biologic treatment of the progressive form of RA.

## INTRODUCTION

The development and progression of rheumatoid arthritis (RA) is associated with several alterations in both the proportions of peripheral blood (PB) Th1, Th17, and Treg cells and their counter-regulatory effects [1–3]. A major role in the pathogenesis of RA is attributed to the immune dysregulation depending on the imbalance between anti-inflammatory Treg cells and pro-inflammatory Th17 cells [1, 2]. The effect on Tregs may be a consequence of the inflammatory conditions in the course of RA, suggesting an impact of the cytokine milieu. Tregs in the presence of a pro-inflammatory environment such as TNF-alpha, IL-6, and IL-1-beta become unstable with respect to the affected forkhead box P3 (Foxp3) gene expression and convert to pathogenic Th17 cells, which expand into the sites of inflammation [4]. In addition, serum IL-6 overexpression in RA is capable of conferring on pathogenic Th17 cells resistance to Treg-mediated suppression [5], thus supporting the shift towards inflammatory conditions. Th17 and Treg cell distribution and function may also be affected by different types of RA treatments [6–8].

In animal model of autoimmune diseases, such as RA, anti-inflammatory action of Th1 cytokines, including IFN-gamma and/or IL-2, has been recently demonstrated [9, 10]. In particular, IL-2 has been suggested to be a cytokine playing a key role in controlling the balance between Treg and Th17 cells in the periphery [10–14]. This Th1 cytokine strongly promotes the differentiation and/or function of Foxp3+ Treg cells, being required for the maintenance of Foxp3 expression by both natural and inducible Tregs [10–13]. It is also responsible for Treg cell survival and homeostasis [14, 15]. Inducible Tregs could differentiate from CD4+CD25- cells in response to IL-2 and TGF-beta [16]. In addition to generation of Tregs, an important aspect of IL-2 function is to constrain IL-17 production by CD4+ T cells, thus inhibiting Th17 polarization [17]. Recently, selective improvement of the levels and function of Tregs has been demonstrated as a result of the low-dose IL-2 immunotherapy in the experimental model of autoimmune disorders [18–23] as well as in the phase I/II clinical trial in patients with type 1 diabetes [24].

In our preliminary data, we reported that the extent of PB Th cell abnormalities and their reversion depended on the duration of the active RA and clearly correlated with progression of the disease [25]. In particular, we found that patients with progressive and, in the most cases, long-term RA remained with quantitative and qualitative Th1 systemic defects as well as a decreased population of functional CTLA-4+ Treg cells in PB despite TNF-alpha inhibitor (iTNF) treatment [25]. Herein, we have extended the study and have performed stimulation assays specific for T cells using anti-CD3 monoclonal antibody to examine the effect of *in vitro* chronic stimulation through the T cell receptor/CD3 complex on the proportions of the Th1, Th17, and Treg cell subpopulations before and after 6 months of treatment with MTX and/or iTNF. Based on our recent demonstration of serum IL-2 shortage during RA progression [25], we decided to verify whether the addition of rIL-2 to anti-CD3 stimulating culture could overcome the observed imbalance between anti- and pro-inflammatory helper T cells. The impact of anti-CD3 ± rIL-2 stimulation is a novelty in RA patients and has provided much information about the reactivity of their PB CD4 T cells to chronic activation either before or after the therapeutic interventions.

## MATERIALS AND METHODS

### Ethics Statement

The study was approved by the local Ethics Committee at Wroclaw Medical University (Poland). According to the 1964 Declaration of Helsinki and its later amendments, written informed consent was obtained from each patient and healthy donor after a full explanation of the procedure.

### Study Populations

The main characteristics of RA patients and healthy volunteers were shown in Table 1. A total of 36 patients diagnosed with RA based on the 1987 revised classification criteria of the American College of Rheumatology (ACR) [26] and 13 healthy individuals were enrolled in the study. The clinical evaluation of RA was based on the medical history, and number of painful and swollen joints; pain intensity was assessed by the patient on a 100-mm visual analog scale (VAS); 28-joint disease activity score (DAS28) was calculated according to the patient as well as physician and laboratory tests (erythrocyte sedimentation rate (ESR), C-reactive protein (CRP)) at the time when the blood samples were obtained. The clinical and laboratory tests were completed before and after 6 months of the therapy.

**Table 1:** The Main Characteristics of RA Patients and Healthy Volunteers

Characteristics	MTX group		iTNF group		Healthy controls
	(*n* = 19)		(*n* = 17)		(*n* = 13)
	Before	After	Before	After	
Age, mean ± SD (years)	54.7 ± 16.4		50.1 ± 6.9		53.5 ± 9.6
Sex, female/male	12/7		14/3		10/3
Duration, mean (range) (months)	15.2 [2.0–79.0]		123.7 [13.0–300.0]		
Steroid, +/−	12/7	7/12	16/1	16/1	
NSAID, +/−	15/4	15/4	13/4	13/4	
Prior DMARDS, +/−	10/9	10/9	16/1	16/1	
DAS28, mean ± SD	5.6 ± 0.9	3.3 ± 1.2	6.2 ± 0.8	4.8 ± 1.1	
RF, positive/negative	16/3	16/3	13/4	13/4	
CRP (mg/l)	24.4 ± 38.1	10.1 ± 26.7	24.4 ± 20.6	19.9 ± 22.4	
ESR (mm/h)	31.2 ± 22.0	18.1 ± 10.5	29.9 ± 16.0	27.2 ± 18.9	

*NSAID* non-steroidal anti-inflammatory drugs, *DMARDs* disease-modifying anti-rheumatic drugs, *DAS28* disease activity score rated by the 28-joint count, *RF* rheumatoid factor, *CRP* C-reactive protein, *ESR* erythrocyte sedimentation rate

All the patients were classified as having active disease if they fulfilled the following criteria: for methotrexate (MTX) treatment, ESR > 30 mm/h and/or CRP > 1.5 mg/dl, DAS28 > 3.2; for treatment with inhibitors of the human tumor necrosis factor alpha (iTNF), ESR > 30 mm/h and/or CRP > 1.5 mg/dl, DAS28 > 5.1. Also, the parameters allowed determination of the improvement according to the criteria suggested by the European League Against Rheumatism (EULAR) [27]: no response (reduction of DAS28 < 0.6), moderate efficacy of the therapy (reduction of 0.6 < DAS28 < 1.2), and good efficacy of the therapy (reduction of DAS28 > 1.2). The other accepted inclusion criteria were as follows: age over 18 years, women and men with reproductive potential had to use reliable contraception, the use of non-steroidal anti-inflammatory drugs (NSAIDs) and glucocorticoids in stable doses was allowed. For the iTNF to be used, treatment failure with at least two traditional disease-modifying anti-rheumatic drugs (DMARDs), including MTX, was required. Therefore, iTNF patients enrolled in the study presented clinical and laboratory signs of advanced and progressive disease, including statistically significant differences in regard to active RA duration (*p* = 0.0000001), DAS28 score (*p* ≤ 0.02), and post-treatment CRP (*p* = 0.05) compared to the MTX group.

In the MTX group, 19 patients with a mean (range) RA duration of 15.2 (2 to 79) months received a stable dose of MTX (10–15 mg once a week orally). If at least moderate improvement was not achieved and there were no significant adverse effects, the dose was up-titrated to a maximum level of 25 mg/week. All MTX patients received 5–15 mg folic acid 24 to 48 h after MTX administration.

In the iTNF group with a mean (range) disease duration of 123.7 (13 to 300) months, patients were administered with recommended doses of iTNF: 3 mg/kg body weight of infliximab, which was given as an intravenous infusion at weeks 0, 2, and 6 and every 8 weeks thereafter (11 patients), subcutaneous injection of adalimumab at 40 mg every other week (two patients), and subcutaneous injection of etanercept at 50 mg every week (four patients). The iTNF patients were allowed to continue treatment with DMARDs; 13 patients with MTX, two patients with sulfasalazine (SSZ), glucocorticoids (prednisone equivalent 10 mg/day), and/or non-steroidal anti-inflammatory drugs, if the treatment regimens were not modified for 4 weeks before the study. The course of the above therapies lasted for at least 6 months.

Among the 19 patients treated with MTX, 14 achieved improvement and 5 a partial response (good and moderate efficacy, respectively). However, among 17 patients who were administered iTNF, 12 achieved improvement, 2 a partial response, and in 3 cases, the treatment was ineffective.

Thirteen healthy controls were free of chronic diseases, including autoimmune, inflammatory, and neoplastic disorders and matched with patients for age and sex with no statistically significant differences in comparison with RA patients.

### Cell Preparation

All blood samples were collected into collection tubes containing 0.2 ml of sodium heparin. Peripheral blood mononuclear cells (PBMCs) were prepared by density gradient centrifugation over Lymphoflot (Biotest, Germany) for further procedures, including stimulation assays and flow cytometric analysis of CD4+ T cell subsets examined.

### Flow Cytometric Analysis of Th1 and Th17 Cells

Before incubation of the cells with phorbol 12-myristate 23-acetate (PMA), CD4+ T cells were purified by negative selection with CD4+ T cell isolation kit by magnetic cell sorting (Miltenyi Biotec) to avoid PMA-mediated internalization and degradation of the CD4 molecule, which would affect the identification of Th1 (CD4+IL-17-IFN-gamma+) and Th17 (CD4+IFN-gamma-IL-17+) cells [28]. Then, the cells were stimulated with 25 ng/ml PMA and 1 μg/ml of ionomycin (Ion) (Sigma-Aldrich) in the presence of 10 μg/ml of brefeldin A (BFA, protein transport inhibitor) and cultured for 4 h at 37 °C in a humidified 5 % CO_2_ incubator followed by the cells’ fixation and permeabilization with BD Permeabilizing Solution 2 (Becton Dickinson) according to the manufacturer’s instruction. Next, the cells were stained with phycoerythrin (PE)-labeled anti-human IL-17 (eBioscience) and fluorescein isothiocyanate (FITC)-labeled anti-human IFN-γ (Becton Dickinson) monoclonal antibodies (mAbs).

### Flow Cytometric Analysis of Treg and Functional CTLA-4+ Treg Subpopulations

Regulatory T cell subsets were defined as CD4+CD25^high^FoxP3+ and CD4+FoxP3+CTLA-4+ cells. Directly after isolation, PBMCs were first aliquoted into tubes without PMA + ion stimulation, and then surface-stained with PerCP anti-human CD4 and FITC anti-human CD25 or FITC anti-human CTLA-4 mAbs. After fixation with 2 % PFA and permeabilization with BD Permeabilizing Solution 2 (Becton Dickinson), the cells were incubated with PE anti-human FoxP3 mAb.

After staining, the cells were washed and immediately analyzed with a FACScan cytometer equipped with Cell Quest software (BD Bioscience Pharmingen). In each case, staining was compared with that of the appropriately labeled isotype control. Lymphocytes were gated on the basis of forward- and side-scatter properties, and at least 30,000 CD4+ T cells were analyzed.

### Activation of Human T Cells *In Vitro*

To determine the effect of *in vitro* stimulation on the populations studied, PBMCs were resuspended to 1 × 10^6^ cells/ml in RPMI 1640 medium (Gibco, Paisley, UK) supplemented with 10 % fetal calf serum (Flow Labs, UK), l-glutamine, and 50 μg/ml gentamycin (Gibco), and cultured with 10 ng/ml of anti-CD3 mAb OKT3 (Ortho, Neckargemund, Germany) in the presence or absence of 500 U/ml of rIL-2 (Eurocetus, Amsterdam, The Netherlands) with subsequent labelling as described above. Control cultures without stimulants were included in each experiment. The cultures were incubated at 37 °C in a humidified atmosphere containing 5 % CO_2_ for 72 h.

### Statistical Analyses

One-way ANOVA test was used to determine significant differences between groups. Paired data were compared by the Wilcoxon signed rank test. Results were considered statistically significant when *p* ≤ 0.05. Data were presented as the mean ± SD. STATISTICA 5.5 (edited in 1999) was used in the statistical calculations.

## RESULTS

### Pre-treatment Distribution of Th1, Th17, and Treg Cells in the Peripheral Blood

**Fig. 1:**
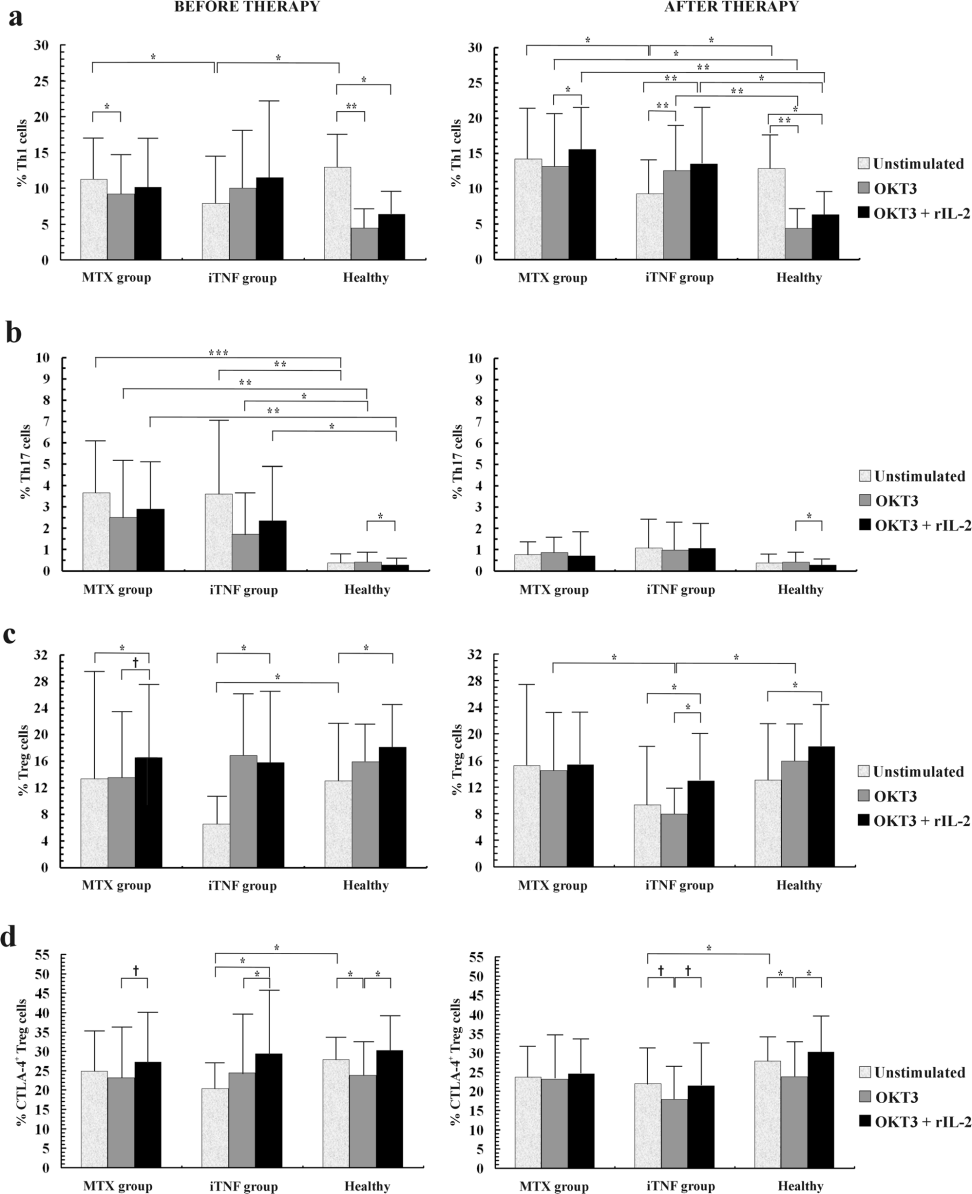
The effect of *in vitro* stimulation with anti-CD3 and anti-CD3 + rIL-2 on helper T cell subpopulations from RA patients before (*left panel*) and after (*right panel*) 6 months of the therapy with MTX and/or iTNF. Results are shown as the mean percentage (mean ± SD) of **a** Th1 (CD4+IL-17-IFN-gamma+) T cells, **b** Th17 (CD4+IFN-gamma-IL-17+) T cells, **c** Treg (CD4+CD25++Foxp3+) cells, and **d** functional Treg (CD4+Foxp3+CTLA-4+) cells. Markers are as follows: ^†^0.05 < *P* ≤ 0.08, *0.001 < *P* ≤ 0.05, **0.0001 < *P* ≤ 0.001, and ***P ≤ 0.0001.

We confirm our recent observation that circulating Th1 cell population was significantly lower in the progressive RA patients compared to the MTX group and controls (Fig. 1a) [24]. PB Th17 populations in both groups of patients were similar and significantly higher than in healthy subjects (Fig. 1b). Compared to controls, Treg cells in patients before iTNF treatment were downregulated, whereas in the MTX group, they reached similar levels (Fig. 1c). Also, circulating CTLA-4+ Treg population in the most advanced RA patients was markedly lower compared to controls (Fig. 1d).

### The Influence of 72-h Culture with Anti-CD3 ± rIL-2 on the Proportions of Th1, Th17, and Treg Cells in RA Patients Before Therapeutics Administration

Anti-CD3 stimulation resulted in a decrease of the Th1 subpopulation in the non-aggressive RA patients (MTX group) and controls, whereas in the iTNF group, the Th1 cell proportion was unchanged. Co-stimulation with rIL-2 did not influence the Th1 subset in patients; in contrast, a marked Th1 decrease was observed in controls. We did not find any differences in the percentages of Th1 cells between studied groups in both culture conditions (Fig. 1a).

We observed that stimulation with anti-CD3 mAb led to no obvious changes in the percentages of Th17 cells in the studied groups. Recombinant IL-2 co-stimulation diminished the Th17 population in controls, only; hence, the Th17 subset remained enriched in all patients at each stimulation condition (Fig. 1b).

Also, anti-CD3 stimulation resulted in no Treg changes in all groups. In contrast, stimulation with anti-CD3 + rIL-2 markedly increased Treg values in all individuals. Considering both stimulating conditions, there were no differences in proportions of Tregs between studied groups (Fig. 1c).

Stimulation with anti-CD3 mAb resulted in a significant decrease of the proportions of CTLA-4+ Treg cells only in controls, whereas there were no changes in RA patients. Co-stimulation with rIL-2 led to increase of the proportions of functional Tregs in progressive RA patients and controls; in MTX patients, its increase was of borderline significance compared to anti-CD3-stimulated cells. In both culture conditions, CTLA-4+ Tregs from all individuals reached similar values (Fig. 1d).

### Post-treatment Distribution of Th1, Th17, and Treg Cells in the Peripheral Blood

We observed no impact of the treatment with MTX and/or iTNF on circulating Th1 cells; hence, the Th1 population remained defective in the iTNF group compared to others (Fig. 1a). We observed a post-treatment decrease of the PB Th17 cell population to normal levels in both groups of patients (Fig. 1b). After iTNF therapy, there was a slight increase of the PB Treg population to a normal level; therefore, no obvious differences in the proportions of PB Treg cells between studied groups were found (Fig. 1c). The treatment did not change the CTLA-4+ Treg subset in all patients; thus, we still observed defective proportions of these functional Tregs in the iTNF patients compared to controls (Fig. 1d).

### The Influence of 72-h Culture with Anti-CD3 ± rIL-2 on the Proportions of Th1, Th17, and Treg Cells in RA Patients After 6 Months of Therapy

Anti-CD3 stimulation resulted in no Th1 cell population changes in the MTX group, its increase in iTNF patients, and a decrease in controls. In all RA patients, the anti-CD3-stimulated Th1 cell population was enriched compared to controls. Co-stimulation with rIL-2 significantly increased the Th1 subset in all RA patients. In contrast, in controls, the addition of rIL-2 led to a decline in the Th1 population. In consequence, the proportions of Th1 cells co-stimulated with rIL-2 in all RA patients were markedly higher than in controls (Fig. 1a).

Also, we found no influence of anti-CD3 as well as anti-CD3 + rIL-2 stimulation on the Th17 population in RA. Co-stimulation with rIL-2 diminished the Th17 subset in controls, only; nevertheless, the *in vitro* stimulated Th17 cell values were comparable in all subjects (Fig. 1b).

**Fig. 2:**
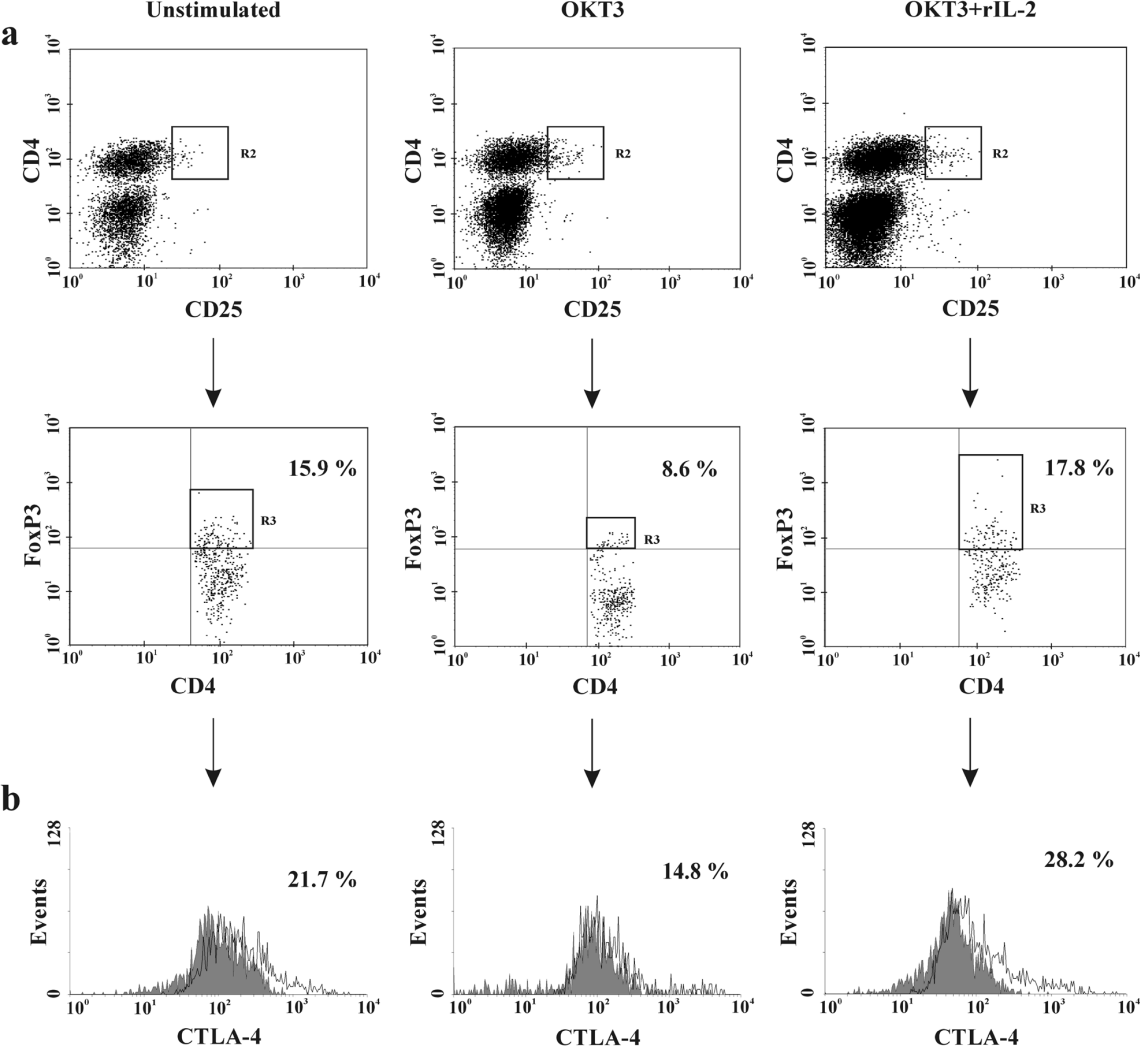
Exogenous IL-2 corrects a defect in the proportion of Treg and CTLA-4+ Treg cells associated with chronic stimulation in progressive RA patients with ongoing iTNF treatment. One representative example of the expression of **a** FoxP3 transcription factor in total CD4+CD25++ T cells (Tregs) as well as the expression of **b** CTLA-4 molecule in Treg cell population (functional Tregs) before (*left panel*) and after 72-h stimulation with anti-CD3 mAb alone (*medium panel*) and with addition of exogenous IL-2 (*right panel*).

Anti-CD3 stimulation did not significantly change the Treg cell population in all individuals; however, a non-significant decrease, revealing a marked Treg defect, was noted in the iTNF group, only. Co-stimulation with rIL-2 resulted in an increase of the Treg population in the iTNF group as well as in controls; thus, co-stimulated Treg values in patients reached a normal level (Figs. 1c and 2a).

We did not find any changes in the percentages of anti-CD3-stimulated CTLA-4+ Tregs in the MTX group. In controls and in the iTNF group, stimulation with anti-CD3 alone diminished the CTLA-4+ Treg population, which reached normal values after co-stimulation with rIL-2. Therefore, the CTLA-4+ Treg proportions after anti-CD3 + rIL-2 stimulation were similar in all individuals studied (Figs. 1d and 2b).

## DISCUSSION

In the present paper, we report that chronic anti-CD3 stimulation possess a normalizing effect on CD4 T cell subpopulations by overcoming Th1 and Treg cell defects seen mainly in progressive disease [25]. With this respect, our finding contradicts the previous demonstration of impaired Th1 responses following stimulation with immobilized OKT3 in RA [29], which may result from substantial differences in the experimental procedures. The mechanisms leading to the partial restoration of both the anti-inflammatory subpopulations under stimulating conditions in advanced RA are not clear. The possibility that Th1 downregulation may become reversible due to the withdrawal from the culture of PB factors which are capable of affecting the Th1 population, such as MTX or statins, cannot be neglected [30, 31]. Moreover, under *in vitro* conditions, selective migration of Th1 cells into the sites of inflammation normally observed in active RA is avoided [32–34]. Therefore, when activated through TCR/CD3, Th1 cells could expand to the normal levels and secrete suitable amounts of IL-2, playing a major role in the generation, survival, and function of Treg cells [10, 13, 17, 35, 36]. In contrast, in untreated patients with non-aggressive RA, anti-CD3 stimulation diminished the Th1 population similarly to the control group, probably due to proper inhibitory function of Tregs and higher consumption of endogenous IL-2 by these cells.

We observed that the only population remaining intact after anti-CD3 ± IL-2 stimulation in untreated RA was Th17, its proportion being similarly expanded in all patients. This effect could be attributed to Th17 resistance to the suppressive function of Tregs and is in agreement with other studies [37–39]. The lack of Th17 normalization might contribute to the maintenance of the inflammation despite reversal of other Th cell populations under stimulating conditions. An unexpected observation, however, was the lack of inhibitory effect of exogenous IL-2 on Th17 differentiation in active disease, which just apparently contradicted previous reports [17, 38]. It is likely that *in vitro* activated PBMCs from patients could secrete into the culture abundant amounts of IL-1, exhibiting opposite and decisive effects on Th17 polarization compared to IL-2 [40, 41]. This finding seems to rule out the clinical relevance of IL-2 supplementation until Th17 normalization. In fact, the reversion of Th17 cell expansion in all RA patients was the main immune advantage of the therapy irrespectively of the therapeutics used. Although the iTNF therapy of the most advanced RA partially corrected also the proportion of Tregs, consistently with our and other reports [7, 25], their qualitative impairment and Th1 systemic defects were still maintained.

In the current study, we demonstrate that therapeutic interventions in RA could change, in addition, the reactivity of CD4+ T cells to stimulation in the disease severity-dependent manner. In particular, we observed that the population of Th1 cells from progressive patients responded to anti-CD3 stimulation more vigorously, which was consistent with the finding of Th1 hyporesponsiveness reversion after iTNF administration [29]. Furthermore, *in vitro* activated Th17 cells remained normal in all patients under the treatment, thus suggesting the long-lasting inhibitory effect of the therapeutics used. The only distinction concerned the Treg cell population from patients with advanced disease, where anti-CD3 stimulation resulted in a marked decrement of Treg population, confronting the lack of such response in both the non-aggressive MTX-treated group and controls. The mechanism underlying the observed downregulatory effect of stimulating conditions on Tregs in the iTNF-treated progressive RA patients is not clear. However, it suggests similarity to the acute responses to the infection agents associated with a limited amount of available IL-2 [42, 43]. The infection-induced Treg cell deficiency found in those studies was essential for the initiation of potent Th1 protective responses in the chronic disease [42, 43]. It cannot be excluded that iTNF administration in the most advanced RA contributes to the development of such a compensating mechanism that includes downregulation of the Treg population. At this stage of the study, it is uncertain whether the activation-induced loss of Tregs in progressive patients is only transient or remains long-lasting, but it should be noted that it might promote the restoration of the anti-inflammatory Th1 population. In fact, when the influence of anti-CD3 stimulation on Th1 cell values was analyzed, a significant impact was found just after the biologic therapy.

When the influence of exogenous IL-2 on the selected CD4+ T cell subsets among the patients under the treatment was analyzed, no significant impact in the MTX group was demonstrated, probably due to the fact that patients with stable RA entered the treatment with no PB Th1 defects. It should be stressed, however, that only in the progressive and iTNF-treated patients, the same who exhibited irreversible systemic loss of IL-2 [25], did supplementation of the culture with this cytokine correct the Treg cell population, which confirms its dependence on the sufficient amounts of IL-2 in the microenvironment. Resistance of IL-2-induced Treg cells to Th17 conversion by IL-6 abundantly concentrated in patients’ sera even after the iTNF administration was demonstrated as well [25, 44]. Recent studies have linked defects in Tregs found both in mouse and human autoimmune disorders to reduced availability of IL-2 [18, 45–48]. As such, IL-2 seems to be a protective rather than pro-inflammatory cytokine that could be involved in the downregulation of chronic inflammation related to RA progression by shifting the balance from Th17-mediated inflammatory conditions to a Treg-mediated tolerant state. Such a normalizing IL-2 potential for effector and regulatory T cell distribution has already been described in the tumor and the infection microenvironment [19–22, 42, 49–57]. In particular, IL-2 administered at low doses to mice [18–22] and humans [53–57] increased the levels of circulating Tregs, improved their regulatory activity, and protected against chronic inflammation, contrasting with the lack of such responses in effector T cells. Our reports confirms that the Treg population rather than effector T cell subset is dependent on IL-2 availability and points to the possibility that low-dose IL-2 immunotherapy may provide a mechanism for recovering the selective expansion of Tregs for the suppression of autoimmune disorders, especially those ongoing with IL-2 systemic deficit. However, the overall reasoning in RA has to be cautious due to the lack of other data on that subject.

In conclusion, our study revealed for the first time that progression of RA is associated with altered responses of Th1, Th17, and Treg cells to stimulation through TCR/CD3. Addition of exogenous IL-2 to the stimulating culture in untreated patients has no considerable impact on the biology of the populations examined. However, in progressive RA patients still lacking IL-2 despite iTNF treatment, it contributes to maintaining a balance between Treg and effector T cells required for the immune control of disease progression. Therefore, our study indicates that exogenous IL-2 may be of potential clinical relevance in the most advanced RA. Further studies on the impact of chronic stimulation with addition of rIL-2 need to be performed to ascertain its usefulness in the treatment of the progressive form of this disease.

## Abbreviations

rIL-2: Recombinant interleukin-2
Th: T helper cell
TNF: Tumor necrosis factor
RA: Rheumatoid arthritis
MTX: Methotrexate
PB: Peripheral blood
Treg: Regulatory T cell
IL-6: Interleukin-6
IL-1: Interleukin-1
Foxp3: Forkhead box P3
TGF: Tumor growth factor
IL-17: Interleukin-17
ACR: American College of Rheumatology
VAS: Visual analog scale
DAS28: 28-joint disease activity score
ESR: Erythrocyte sedimentation rate
CRP: C-reactive protein
EULAR: European League Against Rheumatism
NSAID: Non-steroidal anti-inflammatory drug
DMARD: Disease-modifying anti-rheumatic drug
SSZ: Sulfasalazine
PBMC: Peripheral blood mononuclear cell
PMA: Phorbol 12-myristate 23-acetate
IFN: Interferon
BFA: Brefeldin A
BD: Becton Dickinson
PE: Phycoerythrin
FITC: Fluorescein isothiocyanate
mAb: Monoclonal antibody
CTLA-4: Cytotoxic T-lymphocyte antigen-4
PerCP: Peridinin chlorophyll
PFA: Paraformaldehyde
OKT3: Ortho kung T3
TCR: T cell receptor
